# Bioactive Compound-Fortified Nanomedicine in the Modulation of Reactive Oxygen Species and Enhancement of the Wound Healing Process: A Review

**DOI:** 10.3390/pharmaceutics17070855

**Published:** 2025-06-30

**Authors:** Popat Mohite, Abhijeet Puri, Shubham Munde, Nitin Ade, Aarati Budar, Anil Kumar Singh, Deepanjan Datta, Supachoke Mangmool, Sudarshan Singh, Chuda Chittasupho

**Affiliations:** 1 AETs St. John Institute of Pharmacy and Research, Palghar 401404, Maharashtra, India; mohitepb@gmail.com (P.M.); abhijeetp@sjipr.edu.in (A.P.); shubhamvmunde@gmail.com (S.M.); nitinade2@gmail.com (N.A.); aaratibudar2000@gmail.com (A.B.); 2 Department of Pharmaceutics, United Institute of Pharmacy, Prayagraj 211010, Uttar Pradesh, India; singhanil2682@gmail.com; 3 Department of Pharmaceutics, Manipal College of Pharmaceutical Sciences, Manipal Academy of Higher Education, Manipal 576104, Karnataka, India; deepanjandtt@gmail.com; 4 Faculty of Pharmacy, Chiang Mai University, Chiang Mai 50200, Thailand; supachoke.man@cmu.ac.th; 5 Office of Research Administration, Chiang Mai University, Chiang Mai 50200, Thailand

**Keywords:** bioactive compounds, inflammation, oxidative stress, nanomedicine, nanoparticle, reactive oxygen species, wound healing

## Abstract

Wound healing is a complex biological process that involves the regulation of reactive oxygen species (ROS), which play a critical role in cellular signaling and tissue repair. While the dual nature of ROS means that maintaining controlled levels is essential for effective wound healing, excessive ROS production can hinder the recovery process. Bioactive compounds represent promising therapeutic candidates enriched with polyphenols, which are known for their high therapeutic properties and minimal adverse effects, and are thus highlighted as promising therapeutic candidates for wound healing due to their antioxidant properties. However, their clinical application is often limited due to challenges such as poor solubility and low bioavailability. To overcome this, the encapsulation of these compounds into nanocarriers has been proposed, which enhances their stability, facilitates targeted delivery, and allows for controlled release. The present review highlights emerging innovations in nanomedicine-based drug delivery of natural antioxidants for precise modulation of ROS in wound healing. Moreover, the review elaborates briefly on various in vitro and in vivo studies that assessed the ROS levels using different fluorescent dyes. By modulating ROS levels and improving the local microenvironment at wound sites, these bioactive-nanomedicine formulations can significantly accelerate the healing process of wounds. The review concludes by advocating for further research into optimizing these nano-formulations to maximize their potential in clinical settings, thereby improving therapeutic strategies for wound care and regeneration.

## 1. Introduction

The skin, the body’s largest organ, serves as the first line of defense against environmental conditions, including pathogens, physical injuries, and chemical exposures [1]. When the integrity of the skin is compromised [2], it leads to dermal infections such as wounds. These wounds, whether acute or chronic, present significant clinical challenges [3]. One of the critical factors in wound healing (WH) and the progression from acute to chronic wounds is the generation of reactive oxygen species (ROS). ROS are chemically reactive molecules containing oxygen, such as superoxide anion (O_2_•^−^), hydrogen peroxide (H_2_O_2_), and hydroxyl radical (•OH). They play a dual role in WH [4,5]. At physiological levels, ROS act as signaling molecules that regulate various phases of the healing process, including hemostasis, inflammation, proliferation, and remodeling [6,7]. For instance, ROS are involved in the recruitment of immune cells to the wound site, the activation of signaling pathways that promote cell proliferation and migration, and the formation of new blood vessels (angiogenesis) [4]. However, in chronic wounds, ROS levels are often elevated due to prolonged inflammation, recurrent infections, and impaired antioxidant defenses. This excessive ROS production leads to oxidative stress, damaging cellular components such as lipids, proteins, and DNA. Consequently, oxidative stress can delay the WH process by promoting chronic inflammation, apoptosis, and extracellular matrix degradation. Therefore, modulating ROS levels and mitigating oxidative stress are crucial strategies in wound management [8].

Natural products have emerged as promising candidates for managing dermal infections and promoting WH due to their bioactive properties and ability to modulate ROS levels [9]. Natural products derived from plants, animals, and microorganisms have been used for centuries in traditional medicine to treat various ailments, including skin infections and wounds. These herbal products are rich in bioactive compounds, such as polyphenols, flavonoids, terpenoids, alkaloids, and essential oils, which exhibit antimicrobial, anti-inflammatory, antioxidant, and wound-healing properties [10,11,12]. Bioactive compounds exhibit significant antioxidant activity that mitigates the harmful effects of ROS and assists in WH. In particular, phenolic compounds play a vital role as antioxidants, as they reduce oxidative stress, act as free radical scavengers, and accelerate the WH process. These help in the modulation of inflammatory response in wounds by modulating the cytokine production and immune cell activity. A study demonstrated that the incorporation of cinnamon essential oil into a polymeric composite matrix fused with zinc oxide and mesoporous silica nanoparticles resulted in a multifunctional dressing with both antioxidant and antibacterial properties [13]. This formulation exhibited effective antibacterial activity against pathogens such as *Staphylococcus aureus* and *Escherichia coli*, while maintaining biocompatibility and synergistic performance among nontoxic components. Bioactive compounds often promote the key aspects of cellular repair: collagen synthesis, fibroblast proliferation, and angiogenesis, which ultimately lead to wound closure and tissue regeneration.

Nanomaterials provide several benefits, such as improved bioavailability, targeted distribution, and regulated release of drugs [14]. Nanomedicines can be tailored to target ROS-induced damage and enhance WH when incorporated with bioactive materials [15]. Nanoparticles (NPs) loaded with bioactive compounds such as curcumin, quercetin, or resveratrol can provide sustained and localized antioxidant effects [16,17,18]. Additionally, NPs assist in the stability and bioavailability of antioxidants. To control oxidative stress in wounds, NPs with photothermal or photodynamic features can be used. NPs (such as gold or silver NPs) that absorb light can be used [19]. Photothermal therapy produces heat that can accelerate WH and lessen oxidative stress. ROS-producing NPs can be tailored to target and neutralize excess ROS in chronic wounds, and are used in photodynamic treatment [20]. Both clinical and preclinical studies have demonstrated the potential of bioactive-loaded nanomedicines in the management of ROS-related WH. Clinical trials are increasingly exploring the application of these advanced nanomedicines in human subjects, with promising results in terms of safety, efficacy, and improved WH outcomes [21]. Specifically, a comprehensive literature search was conducted using major scientific databases, including PubMed, Scopus, and Web of Science. The following keywords and their combinations were used: “reactive oxygen species (ROS)”, “bioactive compounds”, “nanomedicine”, and “wound healing”. The search covered publications from 2000 to 2024. Articles were screened and selected based on their relevance to the topic, clinical or preclinical significance, and focus on recent advancements in ROS modulation and wound healing. The review provides summarized information on ROS-generated wounds, their pathogenesis, their measurement, and the ex vivo and in vivo results, with a current update on clinical trials.

## 2. Pathogenies of Wound and the Healing Process

**Figure 1:**
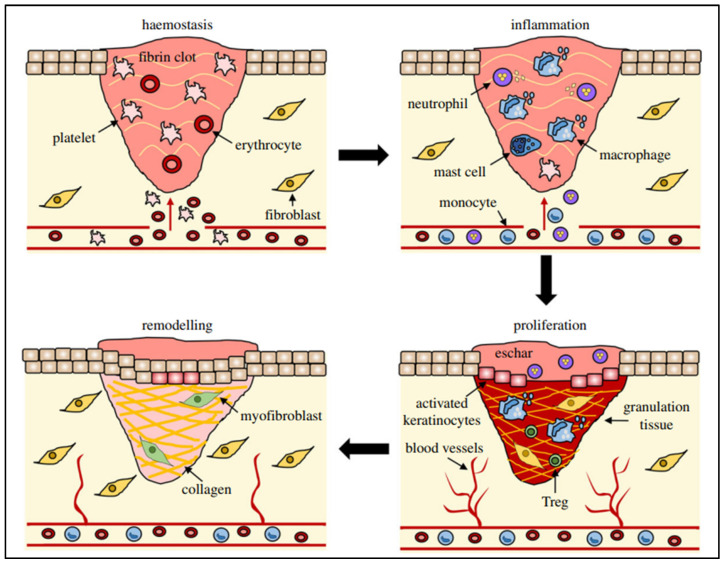
Different stages (hemostasis, inflammation, remodeling, and proliferation) of wound repair and their major cellular components. Reproduced with permission from [30] under the Copyright Clearance Center.

WH is a sophisticated process relying on numerous cellular activities that need to be carefully synchronized to effectively restore injured tissue [22,23]. It is a straightforward sequence where growth factors stimulate cell growth, resulting in a coordinated series of events involving signaling molecules, blood cells, extracellular matrix production, and the proliferation of tissue-specific cells [24]. Wounds can arise due to external or internal factors within an organ, resulting from accidental, intentional, or disease-related events [25]. The process of WH is typically described as four consecutive yet interconnected stages: hemostasis (occurring within hours of injury), inflammation (lasting 1–3 days), proliferation (spanning 4–21 days), and remodeling (extending from 21 days to 1 year) [26,27,28,29] (Figure 1).

The hemostasis stage commences when tissue injury permits blood to seep into the open wound area, initiating the external clotting process and discharging substances that induce localized constriction of blood vessels, such as serotonin [31]. Thrombocytes clump together and initiate blood vessel constriction to diminish blood loss, causing hypoxia, heightened glycolysis, and pH alterations. A clot forms to occupy the wound site, acting as a temporary scaffold for cell migration. Following a 5 to 10 min period of constriction, blood vessels expand, allowing platelets and white blood cells to migrate into the temporary structure. The degranulation of platelets discharges their granules, which contain various growth factors, such as platelet-derived growth factor (PDGF), an activator for cells of mesenchymal origin that stimulates chemotaxis, proliferation, and new gene expression in monocytes, macrophages, and fibroblasts, which play an active role in tissue repair. Insulin-like growth factor-1 (IGF-1) promotes cell proliferation, migration, and differentiation. Epidermal growth factor (EGF) and transforming growth factor-β (TGF-β) regulate the inflammation, angiogenesis, and granulation tissue formation. Further, platelet factor-IV promotes blood coagulation, inhibits angiogenesis, and attracts immune cells to the wound site. These proteins start the process of WH by attracting and activating fibroblasts, endothelial cells, and macrophages [32]. Once haemostasis is accomplished, histamine, released by the activated complement cascade, causes capillaries to dilate and leak, speeding up the migration of inflammatory cells into the wound site and marking the full onset of the inflammatory phase of WH [31].

During the inflammatory stage, there’s a rapid influx of white blood cells (leukocytes) into the wound area, marked by swelling (edema) and redness (erythema). This response typically occurs within the first 24 h and can last up to two days. Immune cells like mastocytes, gamma-delta cells, and Langerhans cells are quickly activated, releasing chemical signals (chemokines and cytokines) to coordinate the immune response [33]. Neutrophil granulocytes are drawn to the wound site by various signals, including extracellular matrix fragments, (TGF-β), complement components (C3a, C5a), and formyl-methionyl peptide bacterial products. Once there, they engulf pathogens through phagocytosis. Macrophages also migrate to the wound area and produce reactive oxygen species (such as superoxide anion) and nitrogen species (like nitric oxide), which are crucial for WH processes [34]. The inflammatory phase of WH continues until all excess bacteria and debris are cleared. However, prolonged inflammation can cause tissue damage and delay healing, leading to chronic wounds. Certain substances, such as lipid oxides and products of arachidonic acid metabolism, have anti-inflammatory properties, helping to regulate the immune response and facilitate the transition to the next phase of healing [35].

Fibroblasts multiply and generate granulation tissue by synthesizing extracellular matrix (ECM) elements like proteoglycans, hyaluronic acid, procollagen, and elastin. This newly formed tissue serves as a conducive base for the development of new blood vessels [36]. The continuous process of depositing and reshaping this matrix is known to be affected by growth factors, including those found in platelet-rich plasma. These growth factors include IL-6 and TGF-β, among others [37]. Stimulating growth factors initiate angiogenesis that enhances the oxygen and nutrient supply to the damaged tissue, facilitating regeneration [34].

The last phase of WH, known as maturation or remodeling, starts about 3 weeks after the injury and can last over a year, depending on the wound. During this phase, natural epithelial growth occurs, and scar tissue matures [38]. In this stage, collagen undergoes reorganization, where type III collagen in the ECM is substituted by type I collagen upon wound closure. Additionally, there is a reduction in the formation of new blood vessels and blood flow. Keratinocyte growth factor (KGF) stimulates keratinocyte proliferation and migration, promotes re-epithelialization, and accelerates wound healing. This process results in the development of a cellular environment and the formation of mature, avascular tissue. Ultimately, the skin can only regain up to 80% of its original tensile strength [39].

The process of healing a wound necessitates the synchronized interaction among the blood supply, dermis, basement membrane, and epidermis. Any disruption in any of these components can potentially prolong the healing process [40]. A chronic wound is one that does not heal in the expected timeframe, typically within 2–4 weeks, despite standard treatment. These wounds are often characterized by an abnormal microenvironment, including issues like edema, reduced blood flow, inflammation, and infection. Healthy individuals usually do not develop chronic wounds; however, certain conditions like diabetes, poor circulation, or immunosuppression can increase the risk [32]. Although the causes may vary at the molecular level, chronic wounds exhibit similar characteristics, such as elevated levels of proinflammatory cytokines, proteases, ROS, and senescent cells. Additionally, they commonly present persistent infections and a lack of functional stem cells [41]. Oxygen (O_2_) is a crucial element needed for generating abundant adenosine triphosphate through mitochondrial processes. Specifically, in WH, it provides the additional energy necessary for tissue regeneration. Nitrogen oxides (NO_X_) play a crucial role in WH by producing ROS, which serve as crucial secondary messenger molecules in this process. Low levels of reactive oxygen are fundamental for intracellular signaling. However, despite their beneficial effects, high concentrations of ROS can be harmful and impede the healing process due to their high reactivity [4]. If the inflammatory phase persists and the level of ROS surpasses the cell’s antioxidant ability, it leads to oxidative stress. This oxidative stress, caused by radical ROS like superoxide anion and hydroxyl radical, as well as non-radical ROS such as H_2_O_2_ and singlet oxygen, can hinder cell movement and growth, leading to tissue harm and prolonged inflammation [42]. Conversely, a deficiency in neutrophils can result in severe infections and hinder proper WH. The role of H_2_O_2_ and other reactive oxygen species in WH. To aid in the process of WH, it is essential to control the elevated levels of ROS that accompany the inflammatory phase. Excessive ROS levels can harm proteins, DNA, lipids, and carbohydrates, leading to oxidative stress, which in turn delays the WH and contributes to scar formation [43,44]. Most patients, especially both diabetic and non-diabetic, with long-term wounds related to oxidative stress, are typically treated with antibiotics, moisture dressings, pressure relief, and surgical removal of the affected area. Recent studies are exploring targeted strategies to enhance the healing rate of chronic wounds, including the use of collagen-derived tissue-engineered grafts, topical growth factors, and various cell types sourced from bone marrow, such as endothelial and epithelial cells. Additionally, effectively regulating the expression of ROS through antioxidants and antioxidative enzymes can substantially mitigate the cellular damage caused by oxidative stress [45].

## 3. Reactive Oxygen Species in Wound Healing

ROS encompass free oxygen radicals, such as superoxide anion radicals (O_2_^•−^) and hydroxyl radicals (^•^OH), along with other oxygen derivatives like H_2_O_2_, hypochlorous acid (HOCl), and singlet oxygen (^1^O_2_), contributing to oxygen radical generation [46]. Together, this set of molecules possesses one or more unpaired electrons, rendering them highly prone to interacting with biological compounds such as proteins, DNA, lipids, and carbohydrates. Various intracellular compartments, including mitochondria, the endoplasmic reticulum, peroxisomes, nuclei, the cytosol, plasma membranes, and extracellular spaces, can produce ROS. In mammalian cells, the mitochondrial electron transport chain is a primary site of ROS generation. Enzymes such as peroxidases, NADPH oxidase, xanthine oxidase, lipoxygenases, glucose oxidase, myeloperoxidase, nitric oxide synthase, and cyclooxygenases catalyze ROS-generating reactions. Externally, factors like air pollutants, tobacco smoke, radiation, dietary components, medications, and environmental toxins can induce oxidative stress. Chemical agents, including quinones, heavy metals (e.g., lead, arsenic), organic solvents, and pesticides, are common external sources of ROS [47]. In the initial inflammatory stage of WH, neutrophils and macrophages migrate into the wound site. Neutrophils, among the primary infiltrating polymorphonuclear leukocytes, play a central role in the inflammatory response at the wound site. When neutrophils are activated, various morphological and metabolic changes occur, all aimed at phagocytosis and the breakdown of foreign substances, resulting in excessive production of ROS to combat infectious threats [48]. The key enzyme responsible for converting molecular oxygen into superoxide radicals is nicotinamide adenine dinucleotide phosphate (reduced form) oxidase, found in both phagosomes and plasma membranes of phagocytic cells. Superoxide radical anions can then be broken down by superoxide dismutase [49] into molecular oxygen and H_2_O_2_. H_2_O_2_, being highly diffusible through biological membranes, may undergo further conversion [50]. Moreover, the NADPH oxidase family can produce ROS through specific enzymes. Nitric oxide enzymes located on cell membranes facilitate electron transfer across biofilms, leading to oxygen reduction and superoxide (O_2_^−^) production. Subsequently, superoxide can undergo chemical reactions to generate various ROS, including H_2_O_2_, peroxyl radicals (HO_2_^−^), and hydroxyl radicals. These ROS serve various functions in cellular processes such as differentiation, proliferation, apoptosis, migration, and contraction [5]. Re-epithelialization, a vital aspect of WH, relies on coordinated cell movement, growth, and specialization. ROS play a significant role in this process by increasing the expression of proteins like collagen, fibronectin, and basic FGF (FGF2) through TGF-1 signaling, promoting fibroblast proliferation and migration. H_2_O_2_ serves as a secondary messenger in WH, influencing the expression of growth factors like heparin-binding EGF-like growth factor and thrombin in vascular endothelial cells. Additionally, H_2_O_2_ can hinder epithelial cell death by regulating vascular endothelial growth factor (VEGF). Remarkably, embryos possess a remarkable ability to swiftly heal wounds without scarring [33]. Nevertheless, excessive production of oxidants, termed oxidative stress, at the wound site invariably hinders the healing process, emphasizing the importance of maintaining a balance between ROS generation and ROS scavenging to facilitate uninterrupted wound closure [51]. At the molecular level, apart from ROS-triggered gene expression that can result in continued pro-inflammatory cytokine release and activation of matrix metalloproteinases, elevated levels of ROS can directly and indirectly alter and break down ECM proteins (potentially via activation of proteolysis) and interfere with the normal function of dermal fibroblasts and keratinocytes [43]. Chronic wounds are frequently marked by either too many ROS or insufficient levels of antioxidant molecules like vitamin E, vitamin C, and glutathione. Interestingly, with aging, the levels of antioxidants in wounds tend to decrease, correlating with slower WH observed in elderly individuals. This indicates that reduced concentrations of antioxidants may lead to delayed WH, as it allows unchecked ROS reactions in the wound, leading to increasing tissue damage over time [52].

Considering the crucial and central role of ROS in the process of wound repair, the precise and accurate detection of these species is absolutely essential for effective treatment. The subsequent section delves into a comprehensive examination of analytical methodologies, which are specifically designed to enable continuous observation of ROS levels both in controlled laboratory environments (in vitro) and within living organisms (in vivo) during the intricate stages of wound healing.

## 4. Analytical Techniques to Measure In Vitro and In Vivo Reactive Oxygen Species

Reactive oxygen species play dual roles in biological systems, acting as signaling molecules at physiological concentrations and causing damage at elevated levels [53]. ROS at stable concentrations can function as signaling molecules to regulate various physiological processes. However, an overproduction of ROS can be harmful, leading to tissue dysfunction or cell death [54]. To study these effects comprehensively, accurate and sensitive analytical techniques are crucial for measuring ROS levels both in vitro and in vivo [55].

### 4.1. In Vitro Analytical Techniques

#### 4.1.1. Fluorescent and Chemiluminescent Probes

A commonly used fluorescent probe is oxidized by ROS into a highly fluorescent form known as DCF (dichlorofluorescein). This probe is especially effective in detecting H_2_O_2_ and hydroxyl radicals [56]. In a study conducted by Huo et al., the fluorescent dye DCFH-DA (dichlorofluorescein diacetate) was employed as a reliable method for detecting ROS in wounded skin cells. The procedure involved culturing the cells, depriving them of serum, and preloading them with DCFH-DA in the dark for 10 min. After removing excess dye through rinsing, the cells were incubated in a medium either with or without epidermal growth factor. Real-time imaging was performed using confocal microscopy with a 488 nm excitation laser, and ROS generation was quantified by measuring fluorescence emission at 522 nm. The fluorescence intensity was expressed as a percentage change over time, with the baseline set at time zero [57]. In another study, Shi et al. incubated telomerase-immortalized human corneal epithelial cells with DCFH-DA to detect intracellular ROS levels. The cells were pretreated with various agents, such as N-acetylcysteine and anti-RAGE antibodies, before incubation with advanced glycation end products. ROS levels were measured using a fluorometer, and accumulation was also visualized with confocal microscopy [58].

Chemiluminescent probes emit light when they react with ROS. These highly sensitive probes are particularly useful for detecting superoxide anions and H_2_O_2_ [59]. Rabbani et al. used L-012 (luminol derivative), a synthetic chemiluminescent probe, to detect superoxide anions and measure ROS in WH studies. In murine models, L-012 allowed real-time, non-invasive imaging of ROS levels in and around cutaneous wounds, helping assess oxidative stress during healing. Its high sensitivity made it ideal for tracking the impact of interventions on redox homeostasis. In wounds, especially in Keap1 knockdown models, it highlighted the role of ROS in delayed healing and enabled continuous monitoring without disturbing the wounds [60]. In another study, lucigenin (2.5 mg/mL) and luminol (10 mg/mL) solutions in saline were prepared and administered intraperitoneally into test animals at doses of 25 mg/kg and 100 mg/kg, respectively, for bioluminescence imaging. Using a spectral system, serial images were captured and analyzed through regions of interest to quantify the total flux. Luminol and lucigenin played distinct roles in detecting ROS: luminol targeted acute inflammation via myeloperoxidase activity in neutrophils, while lucigenin visualized chronic inflammation through NADPH oxidase activity in macrophages. Both probes allowed for noninvasive imaging of inflammation in disease models [61].

#### 4.1.2. Electron Spin Resonance

Electron spin resonance (ESR) combined with spin trapping is a highly specific technique for detecting free radicals. Spin traps such as 5,5-dimethyl-1-pyrroline N-oxide react with ROS to form stable adducts, which can then be detected and quantified using ESR [62]. In an experiment conducted by Ojha et al., electron paramagnetic resonance (EPR) spectroscopy was used to detect ROS in biological systems, specifically in mouse skin wounds, by measuring unpaired electrons in radicals. The spectrometer was tuned for optimal detection of ROS, with parameters including a center field of 44.4 mT and microwave power of 25 mW. Additionally, for spin-trapping experiments, 5,5-dimethyl-1-pyrroline N-oxide was applied to biopsy wounds, and the wound reinstatement was collected and analysed using X-band EPR spectroscopy. This enabled the identification and quantification of ROS, providing valuable insights into oxidative stress during WH. Despite the high precision of electron spin resonance (ESR) in detecting radicals, its clinical usefulness is restricted by factors such as cost, complexity, and limited availability. For real-time monitoring of reactive oxygen species (ROS) within the context of chronic wound models, methods like lucigenin-based imaging demonstrate superior applicability, a point supported by the research documented in the publication [62].

#### 4.1.3. Spectrophotometric Assays

This method measures the reduction of nitro blue tetrazolium (NBT) to formazan by superoxide anions, providing a quantitative measure of superoxide production [63]. Shi et al. employed this assay to evaluate oxidative stress in coronary artery injury. The NBT reduction method measured O^−^_2_ production in tissues. Coronary artery rings were incubated with NBT, which, in the presence of superoxide, was reduced to formazan. The formazan was then extracted, and its absorbance was measured at 540 nm, which demonstrated the level of superoxide produced. The reduction was quantified based on absorbance and incubation conditions [64]. In another study, Siriwattanasatorn et al. evaluated the antioxidant effect of the medicinal plant extract, with propyl gallate as a positive control. In this experiment, NBT reduction was used to measure superoxide production in differentiated HL-60 cells (neutrophils). After treatment with extract and stimulation with PMA, the cells produced superoxide, which reduced NBT to form blue formazan. The formazan was then dissolved in DMSO, and its absorbance was measured at 572 nm, indicating the level of superoxide produced [65].

The Amplex Red/peroxidase assay method quantifies H_2_O_2_ concentration by using the Amplex Red reagent. In the presence of horseradish peroxidase, the reaction produces a fluorescent product that can be measured to determine H_2_O_2_ levels [66]. Basini et al. employed the Amplex Red assay to quantify H_2_O_2_ production in swine granulosa cells exposed to various oxygen conditions. The Amplex Red reagent reacts with H_2_O_2_ to form resorufin, a fluorescent compound. In this study, granulosa cells were cultured under normoxic, hypoxic, and anoxic environments, and after incubation, the fluorescence intensity was measured within cells using a microplate reader, enabling the quantification of H_2_O_2_ levels [67]. Similarly, Hall et al. conducted the Amplex Red assay to quantify H_2_O_2_ concentrations released from SurgihoneyRO™ powders. The assay involved developing a calibration curve with known H_2_O_2_ concentrations. In the procedure, the reagent was dissolved in phosphate-buffered saline to form a stock solution, from which samples were taken at various time points. A reagent master mix containing the Amplex Red substrate and peroxidase was prepared and combined with the SurgihoneyRO™ powder solution or H_2_O_2_ standards in a microplate. After a 30 min incubation in the dark, fluorescence intensity was measured using a plate reader. The resulting fluorescence was proportional to the H_2_O_2_ concentration, which was calculated using the calibration curve and normalized to the assay dilution factor [68].

### 4.2. In Vivo Analytical Techniques

#### 4.2.1. Bioluminescent Imaging

Luciferase-based probes: engineered luciferase enzymes that emit light upon reacting with specific ROS, allowing for non-invasive imaging of ROS in live animals [69].

#### 4.2.2. Magnetic Resonance Imaging

Redox-sensitive magnetic resonance imaging probes: these probes change their relaxation properties in the presence of ROS, enabling the visualization of oxidative stress in tissues [70]. Badr et al. conducted a study outlining a method for in vivo detection of ROS using dynamic nuclear polarization magnetic resonance imaging (DNP-MRI) in a mouse model of plasma-induced skin inflammation. Cold atmospheric plasma was applied to the skin of mice, generating ROS that induced inflammation. DNP-MRI was used to non-invasively monitor redox changes in the skin by injecting TEMPOL, a redox-sensitive contrast agent that reacts with ROS. The MRI signal enhancement allowed visualization of ROS activity and inflammation in real time. The method was validated using EPR to detect hydroxyl radicals, confirming the presence and effect of plasma-generated ROS. This innovative approach demonstrates the utility of DNP-MRI for monitoring ROS-induced redox reactions in live tissues [71]. Similarly, Kawano et al. developed a method for in vivo detection of ROS using DNP-MRI in a mouse model of hepatic fibrosis. The study employed dimethyl nitrosamine to induce liver fibrosis, which resulted in excessive ROS production and a reduction in antioxidant defenses. DNP-MRI, enhanced using a nitrosyl radical probe called carbamoyl-PROXYL, was utilized to non-invasively visualize redox changes in the liver. Following intravenous injection of the probe, DNP-MRI captured changes in signal intensity over time, allowing for the calculation of reduction rates and the generation of redox maps. The results showed slower reduction rates of the probe in fibrotic livers, indicating higher oxidative stress. This method offers a valuable tool for non-invasive monitoring of liver fibrosis and other redox-related disorders [72].

#### 4.2.3. Positron Emission Tomography

In vivo detection of ROS using positron emission tomography (PET) was carried out with the radiotracer [^11^C] hydroethidine ([^11^C] HM) by Alan et al. Following its synthesis through [^11^C] methylation, [^11^C] HM was injected into the bloodstream, where rapid brain uptake was observed. ROS production was induced either systemically by lipopolysaccharide or locally via microinjections of sodium nitroprusside. The retention of the radiotracer in ROS-affected areas was captured through PET imaging, enabling real-time visualization of oxidative stress [73].

Radiolabeled probes: probes like ^18^FSPG, a glutamate-based PET tracer, can indirectly measure ROS by detecting changes in glutathione levels, a key antioxidant [74]. A study conducted by Mota et al., in vivo detection of ROS using PET was performed, which involved synthesizing the ^18^F-labeled tracer 18F-FPBT through nucleophilic fluorination. This tracer, which reacts selectively with superoxide, was then injected into rodent models, such as those with doxorubicin-induced cardiotoxicity. Dynamic PET imaging was conducted to monitor the tracer’s distribution and retention in the body. The oxidized form of the tracer accumulated in areas experiencing oxidative stress, particularly in the heart, allowing for real-time visualization and analysis of ROS activity [74].

#### 4.2.4. In Vivo Fluorescent Imaging

ROS-sensitive fluorescent dyes: dyes such as dihydroethidium can be used for in vivo imaging of superoxide production. These dyes fluoresce upon oxidation by ROS, allowing for real-time monitoring [75]. Similarly, DCFH is a widely used small-molecule fluorescent dye for studying ROS signaling and oxidative stress in plant cells and green algae. As a member of the reduced fluorescein group, its diacetate ester form (DCFH-DA) is cell-permeable, allowing for the accumulation of non-fluorescent DCFH, which is then oxidized by H_2_O_2_ and other oxidants to produce the fluorescent product 2′,7′-dichlorofluorescein. This fluorescence can be easily monitored via fluorescence microscopy and flow cytometry instruments. Thus, the DCFH-DA is highly useful for detecting both intracellular and extracellular ROS generation during oxidative stress and damage [76].

## 5. Bioactive Compound Capability in Reducing Oxidative Damage Linked to ROS

Oxidative stress, caused by an imbalance between ROS and the body’s antioxidant defenses, significantly contributes to various diseases. This imbalance leads to cellular damage, inflammation, and the advancement of chronic conditions such as diabetes, neurodegenerative disorders, and cancer. Recently, the potential therapeutic benefits of bioactive compounds in mitigating oxidative damage have attracted considerable interest. Various phytochemicals, such as curcumin, quercetin, resveratrol, epigallocatechin gallate, apigenin, sulforaphane, and ursolic acid, have been shown to effectively modify nuclear factor erythroid 2-related factor-2 (Nrf2) signaling and prevent a range of diseases [77,78]. This section investigates the bioactive compounds that have shown significant efficacy in reducing oxidative stress in both in vitro and in vivo studies.

### 5.1. Curcumin

**Figure 2:**
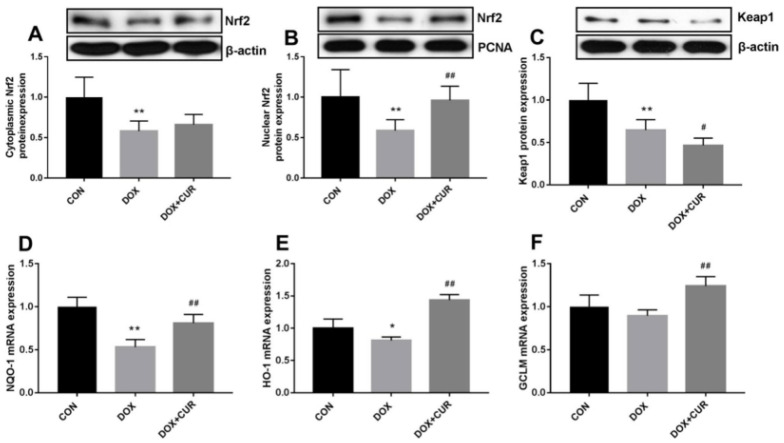
Effects of CUR on the activation of Keap1-Nrf2 in DOX-treated rats. Protein expression of Nrf2 in the cytoplasmic (**A**), protein expression of Nrf2 in the nuclear (**B**), protein expression of Keap1 (**C**), mRNA expression of NOQ-1 in the hippocampus (**D**), mRNA expression of HO-1 in the hippocampus (**E**), mRNA expression of GCLM in the hippocampus (**F**). * *p* < 0.05 and ** *p* < 0.01 compared to the control group. ^#^ *p* < 0.05 and ^##^ *p* < 0.01 compared to the DOX group. Reproduced with permission from [84] under the Creative Commons CC BY 4.0.

**Figure 3:**
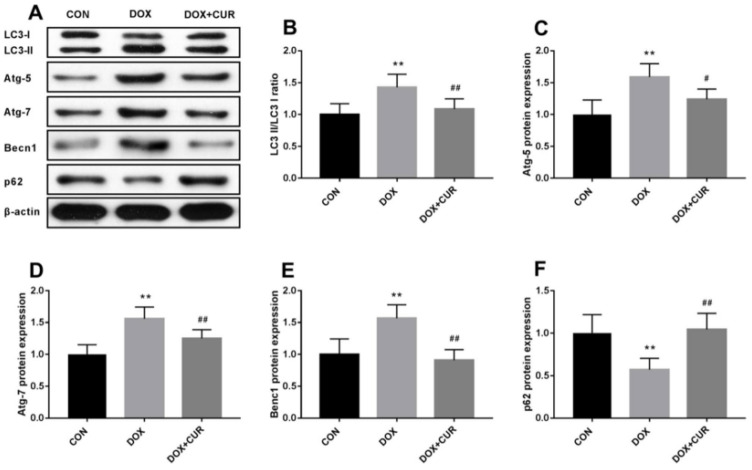
(**A**) Effects of CUR on DOX-induced autophagy in the hippocampus. LC3-II/LC3-I ratio (**B**), protein expression of Atg-5 (**C**), protein expression of Atg-7 (**D**), protein expression of Becn1 (the label in figure E is incorrect; this is a typing error) (**E**), protein expression of p62 (**F**). ** *p* < 0.01 compared to the control group. ^#^ *p* < 0.05 and ^##^ *p* < 0.01 compared to the DOX group. Reproduced with permission from [84] under the Creative Commons CC BY 4.0.

**Figure 4:**
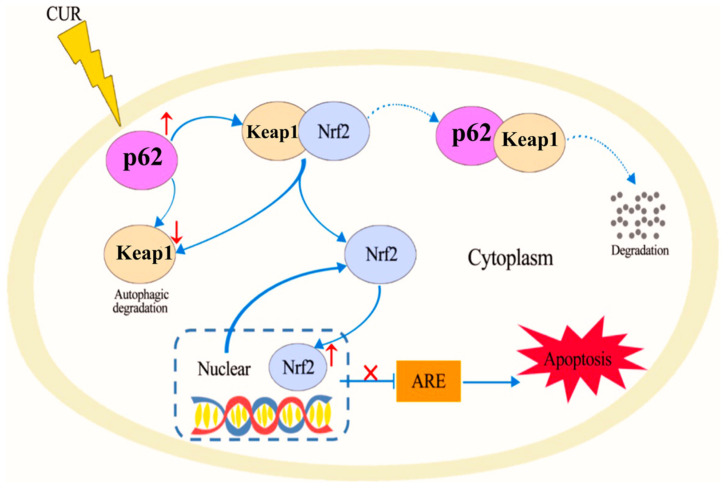
Proposed mechanisms of CUR in protection against DOX-induced neurotoxicity. Reproduced with permission from [84] under the Creative Commons CC BY 4.0.

Curcumin [79], a polyphenolic compound derived from the turmeric plant, has attracted considerable interest for its potential health benefits, especially for treating oxidative stress and related diseases [23,80,81]. It has been shown to activate the Nrf2/antioxidant response element signaling pathway effectively, which in turn enhances the expression of several antioxidant enzymes. These enzymes are essential in protecting cells from oxidative damage by eliminating ROS and mitigating their harmful effects [82]. In addition to its antioxidant properties, curcumin also inhibits inflammatory mediators. This dual action reduces oxidative stress while simultaneously lowering inflammation, making curcumin a potent candidate for preventing and managing oxidative stress-mediated diseases [83]. He et al. demonstrated that CUR can effectively provide protection against doxorubicin (DOX)-induced oxidative stress and neurotoxicity through two key mechanisms: activating the Keap1-Nrf2-ARE antioxidant pathway and regulating autophagy. DOX treatment modulates the oxidative stress in neurons, as shown by increased oxidative markers like 4-HNE and Malondialdehyde (MDA) and reduced levels of antioxidant enzymes such as catalase (CAT) and glutathione peroxidase (GPx) in the hippocampus. CUR counters these effects by reducing these markers and restoring antioxidant enzyme activity. Mechanistically, Figure 2 illustrates that CUR activates the Nrf2-ARE pathway by dissociating Nrf2 from its inhibitor Keap1, allowing Nrf2 to enter the nucleus and stimulate antioxidant gene expression, including heme oxygenase-1 (HO-1) and NADPH quinone dehydrogenase 1 (NQO1), which neutralize oxidative damage. Additionally, Figure 3 reveals that DOX exposure triggers excessive autophagy, a cellular self-repair mechanism, leading to potential cellular damage if unregulated. CUR alleviates this by reducing autophagy markers (LC3-II/LC3-I ratio, Atg5, Atg7, Beclin-1), thus preventing autophagy overload and stabilizing cellular functions while lowering ER stress. The integrated mechanisms in Figure 4 tie together these pathways. CUR’s combined activation of the Keap1-Nrf2-ARE pathway and its modulation of autophagy via the p62-Keap1-Nrf2 feedback loop enhance cellular defenses, thereby reducing oxidative damage, endoplasmic reticulum stress, and neurotoxicity. This multi-targeted approach provides CUR’s neuroprotective effects against DOX-induced toxicity and associated depressive-like symptoms in animal models [84].

**Figure 5:**
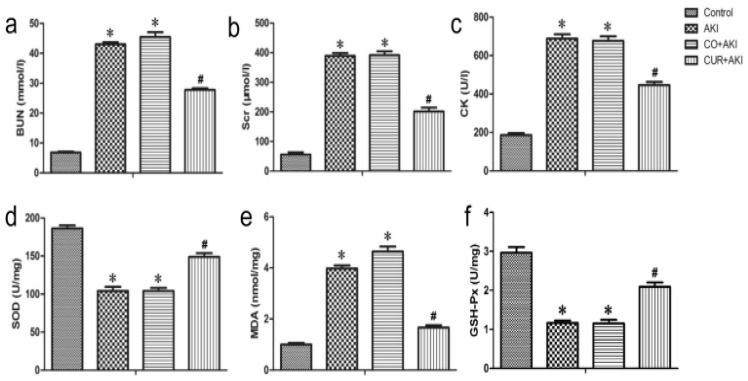
Effect of curcumin on serum BUN, Scr, CK, and kidney SOD, MDA, and GSH-Px in rats. Curcumin treatment on renal tissue ameliorates glycerol-induced acute kidney injury. (**a**–**c**) Compared with the control group, curcumin significantly reduced Scr, BUN, and CK levels 72 h after glycerol injection. Statistical significance: * *p* < 0.01 versus the control group; ^#^ *p* < 0.01 versus the AKI and CO + AKI groups, respectively (n = 12). Data are represented the means ± SD. (**d**–**f**) Curcumin significantly reduced SOD and GSH-Px levels compared with the control group and markedly increased MDA compared with AKI and CO + AKI groups 72 h after glycerol injection. Each bar represents the mean ± SD (n = 12). Statistical significance: * *p* < 0.01 versus the control group; ^#^ *p* < 0.01 versus the AKI and CO + AKI group. Reproduced with permission from [85] under the Creative Commons CC BY 4.0.

Wu et al. reported that CUR effectively inhibited oxidative stress and reduced renal injury and apoptosis in rhabdomyolysis-induced acute kidney injury by activating the AMPK, Nrf2/HO-1, and phosphoinositol 3-kinase (PI3K)/Akt signaling pathways. In Figure 5, biochemical analysis shows that CUR significantly increased levels of antioxidant enzymes such as SOD and GPx, while reducing MDA, a marker of oxidative stress. CUR upregulates Nrf2 and HO-1 expression in renal tissue. Nrf2 activation allows cells to mount a defense against oxidative damage by increasing HO-1, which helps to eliminate ROS and reduce cellular stress. This mechanism underlies curcumin’s ability to attenuate kidney injury and support cellular protection against oxidative stress [85].

### 5.2. Quercetin

**Figure 6:**
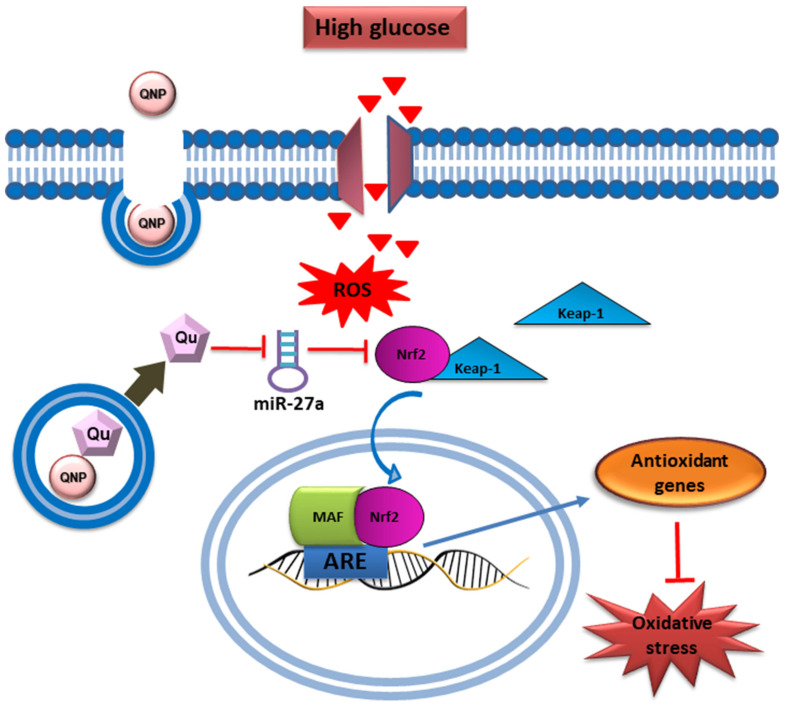
Schematic presentation of the beneficial effect of quercetin conjugated with superparamagnetic iron oxide NPs on miR-27a/Nrf2-dependent antioxidant pathway in the hippocampus of diabetic rats. Reproduced with permission from [89] under the Creative Commons CC BY 4.0.

Quercetin, a powerful antioxidant flavonoid present in various fruits, vegetables, and grains, is known for its effectiveness in reducing oxidative damage caused by ROS [86]. It affects several kinase pathways, such as GSK3β, PI3K/AKT, and MAPK, which are involved in regulating Nrf2 signaling. By modulating these pathways, quercetin enhances the antioxidant response, offering additional protection against oxidative damage [87]. Similar to other phytochemicals, quercetin may impact gene expression related to antioxidant defense through epigenetic mechanisms. This effect leads to the continuous activation of Nrf2, ensuring long-term protection against oxidative stress. According to L. Zhang et al. [88], non-communicable diseases (NCDs) exhibit a significant correlation with oxidative stress and increased reactive oxygen species (ROS). Quercetin, a flavonoid found in nature, reduces the levels of ROS and has strong antioxidant capabilities. It instigates the Nrf2 signaling pathway, resulting in an enhanced defense against oxidative damage within the body. Quercetin also enhances mitochondrial function and mitigates inflammation through Nrf2 modulation. Quercetin demonstrates promise as a therapeutic agent for treating NCDs due to these characteristics. Further, Ebrahimpor et al. showed that in streptozotocin-induced diabetic rats, quercetin-conjugated superparamagnetic iron oxide NPs lowered miR-27a levels and enhanced the expression of Nrf2 and its downstream genes, such as SOD, GPx, and CAT. Figure 6 illustrates how quercetin-conjugated superparamagnetic iron oxide NPs enhance the Nrf2-dependent antioxidant pathway by reducing the expression of miR-27a. In diabetic rats, upregulation of miR-27a typically suppresses Nrf2, resulting in decreased antioxidant enzyme levels, including SOD1 and CAT. Quercetin-conjugated superparamagnetic iron oxide NPs, however, downregulate miR-27a, enabling Nrf2 activation, which subsequently increases the expression of antioxidant enzymes. This cascade helps reduce oxidative stress and mitigate diabetes-induced memory impairment, underscoring quercetin’s therapeutic potential in oxidative stress management [89].

### 5.3. Resveratrol

Resveratrol is a naturally occurring polyphenolic compound that is found in several plants, notably grapes, berries, and peanuts. It possesses diverse biological properties, including the ability to act as an antioxidant, reduce inflammation, and inhibit platelet aggregation [77]. With three separate hydroxyl groups, resveratrol’s main antioxidant activity is linked to its capacity to scavenge ROS [90]. Hui et al. found that resveratrol exerts neuroprotective effects by activating the PI3K/Akt/NRF2 signaling pathway and increasing the expression of HO-1 in PC12 cells with amyloid-beta Aβ1-42-induced cytotoxicity. The mechanism behind the process is that resveratrol activates the PI3K/Akt pathway, leading to phosphorylation of Akt, which then promotes Nrf2 translocation into the nucleus. In the nucleus, Nrf2 binds to the antioxidant response element, inducing HO-1 expression. HO-1 plays a protective role by degrading heme into biliverdin, carbon monoxide, and free iron, which collectively help to combat oxidative stress. This mechanism not only reduces oxidative stress markers like ROS and malondialdehyde but also increases antioxidant levels such as GSH and SOD, providing neuroprotection against Aβ1-42-induced cytotoxicity [91].

Kim et al. reported that while SIRT1/AMPK and PPARα signaling pathways were downregulated in a mouse model of age-related renal injury, resveratrol improved renal function, reduced proteinuria, and lessened glomerulosclerosis by activating Nrf2 signaling and enhancing SIRT1/AMPK and PPARα pathways in the kidneys. Resveratrol treatment increases the nuclear translocation of Nrf2, promoting the expression of antioxidant enzymes like HO-1 and NQO-1, which protect against oxidative damage. Additionally, resveratrol boosts SIRT1 activity, which supports mitochondrial function and further reduces oxidative stress. Together, these pathways combat oxidative stress in the kidneys, alleviating age-related renal damage and improving kidney function in aging mice. This mechanism highlights resveratrol’s therapeutic potential for managing oxidative stress-induced damage in aging tissues [92].

### 5.4. Epigallocatechin-3-Gallate

Epigallocatechin-3-gallate (EGCG), a major polyphenol in green tea, is well known for its health-promoting effects, particularly in reducing oxidative stress through the modulation of the Nrf2 signaling pathway. Evidence from various in vivo models has demonstrated that EGCG has protective effects against cancer, cardiovascular diseases, and neurodegenerative disorders. These studies reveal that EGCGs exhibit the potential to reduce oxidative damage and improve health outcomes in these conditions [93]. In a study, Han et al. found that according to the neurological score, EGCG demonstrated neuroprotective effects by improving cerebral functions, decreasing ROS generation, and activating the Nrf2/ARE signaling pathway. In another study, EGCG-treated rats showed reduced brain infarction and improved neurological function after ischemia-reperfusion injury. This protection arises from EGCG’s ability to increase Nrf2 expression, which subsequently enhances downstream antioxidant enzymes like heme oxygenase-1 and glutamate-cysteine ligase, essential in managing oxidative stress. In addition, Kanlaya et al. reported that EGCG reduces oxidative stress in kidney diseases by activating the Nrf2 pathway and limiting inflammation. EGCG interrupts the interaction between Nrf2 and its inhibitor, Keap1, allowing Nrf2 to translocate to the nucleus and bind to antioxidant response elements in the DNA. This binding triggers the expression of protective enzymes like HO-1 and GPx, enhancing cellular defenses against oxidative damage [94]. In a study on human corneal epithelial cells (HCECs) exposed to oxidative stress, the combination of quercetin-loaded PLGA nanoparticles and EGCG significantly reduced ROS levels, with hydrogen peroxide and superoxide anion production decreasing by 57.27% and 41.28%, respectively. Moreover, at a low concentration (2.6 µM), this combination exhibited ROS inhibition comparable to 5 mM N-acetyl-L-cysteine (NAC), a well-established antioxidant. These findings suggest that EGCG-quercetin co-administration may offer enhanced therapeutic benefits for oxidative stress-related diseases. The sustained release of these polyphenols through nanoparticle-based delivery further amplifies their bioavailability and efficacy, making them promising candidates for future antioxidant therapies [95].

### 5.5. Gallic Acid

**Figure 7:**
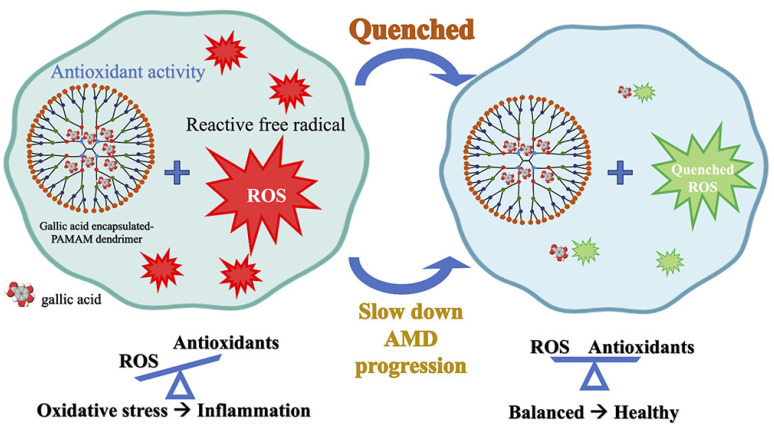
Gallic acid encapsulated within poly(amidoamine) dendrimers to enhance its stability, controlled release, and bioavailability. Reproduced with permission from [96] under the Creative Commons CC BY-NC-ND 4.0.

Gallic acid, a well-known polyphenolic antioxidant, plays a crucial role in reducing ROS and mitigating oxidative stress-related cellular damage. In retinal pigment epithelial (ARPE-19) cells, which are particularly susceptible to oxidative stress, gallic acid has demonstrated protective effects against ROS-induced damage. However, despite its strong antioxidant potential, its low bioavailability and instability under physiological conditions limit its therapeutic applications. To address these limitations, researchers have encapsulated gallic acid in poly(amidoamine) (PAMAM) dendrimers to enhance its stability, controlled release, and bioavailability (Figure 7). The study compared two formulations, G4(OH)-Ga and G5(OH)-Ga, which significantly improved gallic acid’s radical scavenging activity. Antioxidant evaluations using DPPH, ABTS, and FRAP assays revealed that encapsulated gallic acid exhibited stronger ROS-scavenging effects than its free form, suggesting that encapsulation allows for a more sustained and controlled antioxidant effect. Further investigations demonstrated that gallic acid, both in free and encapsulated forms, effectively inhibited H_2_O_2_-induced ROS production in ARPE-19 cells. Interestingly, PAMAM dendrimers alone showed no significant ROS reduction, confirming that the antioxidant properties were solely attributed to gallic acid. The encapsulation process not only enhanced ROS scavenging efficiency but also improved the stability of gallic acid under physiological conditions. In particular, the sustained release profile observed in G5(OH)-Ga allowed for prolonged antioxidant activity, ensuring better protection against oxidative stress [96].

## 6. Bioactive Compound-Fortified Nanomedicine in the Modulation of ROS

**Figure 8:**
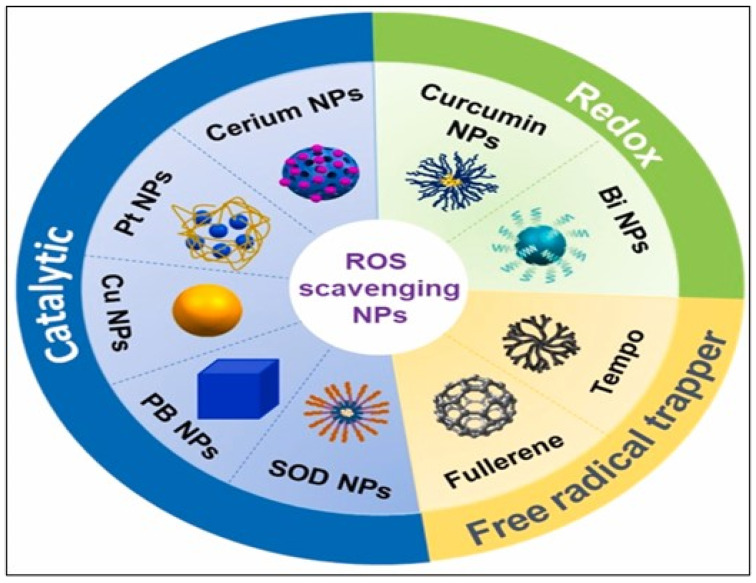
Bioactivities of representative inorganic nanoparticles used in wound healing. Note: The term “Cerium” as labeled in the original figure refers specifically to cerium oxide (CeO_2_ or Ceria). Reproduced with permission from [97] under the Creative Commons CC BY-NC-ND license.

Nanomedicines fortified with bioactive compounds play a crucial role in modulating ROS, which are involved in various diseases [86]. Breakthroughs in nanotechnology, particularly in nano-chemistry and nanomanufacturing, have revolutionized the pharmaceutical and biotechnology fields, leading to significant advancements in antioxidant therapy. Nanomaterials with distinctive ROS-scavenging properties, including nanoparticulate carbon, selenium, cerium, platinum, copper, redox polymers, and polyphenols, have been developed to moderate inflammatory ROS responses [97]. Research shows that these nanomedicines enhance the delivery and effectiveness of bioactive compounds, leading to improved antioxidant activity and reduced oxidative stress. For example, a study by Xin et al. demonstrated the potential of nanocarriers to encapsulate antioxidants, thereby increasing their bioavailability and therapeutic effects against ROS-induced damage [98]. Another study highlights the synergistic effects of combining bioactive compounds with nanotechnology. This combination not only enhances the stability of the compounds but also enables targeted delivery to affected tissues, further modulating ROS levels [99]. Figure 8 illustrates bioactive compound-fortified nanomedicine in the modulation of ROS.

Nanomedicines overcome the limitations of conventional drug delivery systems. Particularly, NPs can be engineered to encapsulate and deliver the bioactive compound with a smaller particle size, high precision, and therapeutic index [100]. There are different types of nanomedicines used for WH. These nanoparticulate systems are designed to target the wound tissues, with localized action and controlled release [101].

Polymeric nanocarriers are a versatile and widely used platform in wound healing due to their ability to encapsulate and deliver hydrophilic and hydrophobic bioactive compounds. These systems have revolutionized the delivery of therapeutic agents by offering controlled and sustained release profiles, enhancing therapeutic efficacy and reducing the frequency of administration [102]. PLGA-based nanoparticles have been extensively investigated for their potential in delivering bioactive compounds such as quercetin, which is highly beneficial in wound healing. Encapsulation within PLGA nanoparticles improves solubility and facilitates sustained release, ensuring a constant therapeutic level at the wound site [103]. Chitosan-based nanocarriers represent another significant advancement in polymeric wound healing systems, as they can encapsulate a variety of therapeutic agents, including antibiotics, growth factors, and antioxidants [104]. Solid lipid nanoparticles loaded with curcumin have shown significant promise in overcoming the bioavailability issues associated with curcumin. Incorporating curcumin into SLNs not only protects it from degradation but also ensures a sustained and localized release at the wound site. When integrated into hydrogels, curcumin-loaded SLNs enhance skin penetration and provide prolonged therapeutic action, significantly improving wound closure rates and reducing inflammation [105]. Zein-based nanofibers incorporating *Moringa oleifera* extract exemplify the use of natural polymeric systems in wound care [106]. Zein, a hydrophobic protein derived from corn, is biodegradable, biocompatible, and capable of forming fibrous structures that mimic the extracellular matrix. The electrospinning of zein with *Moringa oleifera* extract results in nanofibrous mats that sustain the release of bioactive compounds, reduce oxidative stress, and prevent microbial colonization, thereby enhancing the overall wound healing process. Polymeric nanocarriers offer numerous additional advantages, including the ability to tailor their size, surface charge, and drug release profiles [107]. However, the clinical translation of polymeric nanocarriers faces several challenges, including the need for scalable and reproducible manufacturing processes, comprehensive biocompatibility assessments, and regulatory approvals.

Natural compound-stabilized nanocarriers have gained attention in wound healing due to their bioactivity, biocompatibility, and environmentally friendly synthesis routes. These nanocarriers utilize plant-derived polyphenols, flavonoids, and other bioactive phytochemicals for their antioxidant, anti-inflammatory, and antimicrobial properties [108]. Green synthesis of silver nanoparticles using plant extracts rich in polyphenolic compounds is a promising approach, as it enhances ROS scavenging capabilities and reduces cytotoxicity compared to chemically synthesized nanoparticles. The polyphenolic coating on these nanoparticles improves their stability and modulates their interaction with biological tissues, promoting wound healing while minimizing side effects [109]. Ficus trijuga-loaded lipid nanocapsules and grape seed extract-conjugated nanoparticles are also examples of natural compound-based systems with significant wound healing potential [110]. These systems provide a sustained release of bioactive agents, ensuring prolonged therapeutic action at the wound site, minimizing the need for frequent dressing changes and patient discomfort. Combining these nanocarriers with polymeric matrices like hydrogels or nanofibers can further enhance their therapeutic efficacy. These systems represent the three pillars of nanotechnology-driven wound healing, each offering unique advantages in terms of ROS modulation, bioactive compound delivery, and wound healing enhancement.

Topical formulations such as creams, gels, films, and patches play a vital role in delivering the therapeutic agent to improve skin conditions and disorders. The most significant advantage of incorporating NPs into topical formulations is the enhancement of drug delivery due to their small size, which can penetrate the stratum corneum more effectively than conventional drug delivery systems [111,112]. Additionally, nanoparticulate systems are able to encapsulate both hydrophilic and hydrophobic drug systems that are not compatible with conventional formulation. Various studies have been reported on the incorporation of bioactive-mediated NPs for WH [113]. Abdollahi et al. developed hydrogels based on sodium carboxymethyl starch incorporating copper nanoparticles as a WH composite. They evaluated its antioxidant and antimicrobial properties, revealing that the nanocomposite exhibited approximately 75% antioxidant activity, indicating its strong potential for ROS scavenging at the wound site [114]. Sandhu et al. formulated CUR-loaded solid lipid nanoparticles (SLNs) incorporated into a hydrogel for wound healing applications. Their findings demonstrated that the composite exhibited significant antioxidant activity and effectively reduced the expression of pro-inflammatory cytokines [115]. Hashad et al. prepared lipid nanocapsules loaded with Ficus trijuga extract, which is rich in flavonoids and triterpenoids known for their antioxidant and anti-inflammatory properties. The wound healing activity was evaluated in a rat model, and the findings revealed a 2.62% improvement in wound closure compared to the standard treatment [116]. Mamgain et al. developed zein-based nanoparticles incorporating Moringa oleifera extract, which contains a wide range of bioactive compounds. The formulation demonstrated enhanced wound healing activity compared to the standard treatment [107]. Fereydouni et al. fabricated zein-based nanofibers incorporating 3% and 5% cerium oxide nanoparticles and evaluated their cytotoxicity, intracellular ROS levels, and anti-bacterial activity for wound healing applications. The results revealed that the nanofiber composites reduced intracellular ROS levels and promoted the proliferation of human dermal fibroblast cells [117].

Nalini et al. developed a carbopol nanogel formulation of quercetin incorporated with alginate/chitosan NPs in varied concentrations for the management of wounds. The in vitro release study demonstrated a 62.51% of sustained release over a period of 24 h. Moreover, in vivo acute dermal toxicity showed insignificant toxicity for the test formulation compared to the control in the tested rats. Moreover, antioxidant-based assay such as SOD, CAT, LPO, and NO indicated enhance free radical scavenging ability of test formulation. Furthermore, the test formulation showed re-epithelialization of treated rat skin in a shorter time compared to animals treated with quercetin alone. Additionally, histopathological results evidence improved re-epithelization and collogen formation by the deficit of inflammation, established fibrous tissue, well-organised fibroblasts, and blood capillaries, suggesting quercetin nanoparticles exhibit synergistic wound healing capabilities [118]. In a similar study, quercetine-loaded chitosan tripolyphosphate NPs significantly accelerated the cutaneous wound healing in Wistar rats. These results were further supported by the biochemical assays, where expressions of interleukin 10, vascular endothelial growth factor, and transforming growth factor-beta 1 were significantly increased with treatment of test nanoparticles. Moreover, the histopathological revealed increased blood vessel density, decreased inflammatory cells, increased number of myofibroblasts, deposition and arrangement of collagen fibers, and re-epithelialization on treatment with quwecwtine loaded chitosan nanoparticles [119].

Galic acid is another known bioactive compound that possesses excellent antioxidant potential with other biomimetic attributes. However, gallic acid in liquid form is generally unstable and tends to degrade, particularly due to variation in temperature, oxygen, and light. Yan et. al., fabricated gallic acid nanocrystals were prepared using “Top-down” techniques and fortified within a polyacrylic acid matrix to form a hydrogel via co-gelation, exhibiting good antibacterial activity and scavenging efficiency. The in vivo study demonstrated accelerated wound healing by promoting neovascularization, epidermal regeneration, and collagen deposition. The results suggested that formulated nanocrystals fortified hydrogel could be a multifunctional alternative to potent antibiotics for effective wound treatment in clinic [120].

Epigallocatechin gallate (EGCG) exhibits a robust intracellular ROS scavenging ability were transformed to nanopaticel form via self-assembly. The fabricated NPs demonstrated better cellular uptake, significantly enhancing their biocompatibility, intracellular ROS scavenging capacity, and ability to mitigate DNA damage. Moreover, EGCG-NPs facilitated fibroblast proliferation and migration, while inhibiting the polarization of RAW 264.7 cells towards the M1 phenotype in vitro. Additionally, EGCG-NPs exhibited markedly improved radioprotective efficacy over free EGCG, effectively reduced skin edema and ulceration, allevated pathological conditions such as interstitial edema, dermal fluid accumulation, and inflammatory infiltration, with decrease in duration of skin injury, and promoting wound healing [121]. The anti-xidative and anti-inflammatory properties of resveratrol have been proven in several literatures. However, the resveratrol efficacy is non-selective and high leves of resveratrol may inhibit cell growth and promote oxidation. In an attempt by Tang and co-worker, developed resveratrol NPs fucntionalized by phenylboric acidm with an improve solubility and antibacterial activity. The fabricated NPs demonstrated downregulation of inflammatory cytokines expression and reduced intracellular excessive ROS significantly than those tested native resveratrol. Furthermore, the NPs incoporated gel significantly accelerated the formation of granulation tissue, collagen depostion, and re-epithelization, facilitating improved wound healing efficacy [122].

A new potent bioactive compound rhodomyrtone, originating from *Rhodomyrtus tomentosa* have been reported to demonstrate significant antiinflammatory acitvity both in pure form and *R. tomentosa* leaf extract incorporated form [12,123]. Chorachoo et al., tested the efficacy of rhodomyrtone for curtailing TNF/IL-17A driven inflammation. The results suggested that on stimulating human skin organ cultures with TNF/IL-17A to represent skin inflammation, rhodomyrtone can significantly decereased inflammatory gene expression and the expression and secreation of inflammatory protiens. Followed to this Wunnoo et. al., transformed rhodomyrtone to vesciular drug delivery system and tested efficacy clinically, suggesting that serum fortified with bioactive compound is safe and potentially effective in the management of inflammatory lesions [124]. While Ontong et. al., prepared rhodomyrtone-rich extract using microwave assisted technology and transform to a topical formulation and tested the efficacy clinically. The results suggested here too that the formulation can be effectively employed as topical inflammatory agent [125]. Several reports indicate that anti-inflammatory agent and ROS are closely linked, with ROS playing a significant role in both inflammation and mechanisms of action of many anti-inflmmatory drugs [6]. Therefore, the bioactive compounds that act as potent anti-inflammatory agent can also balance the ROS and regulate the wound healing process [7]. Summary of nanocarriers incorporated potent bioactive compounds for wound healing applications is presented in Supplementary Table S1.

## 7. Challenges and Future Prospects

The integration of bioactive compounds into nanocarriers not only improves the bioavailability and efficacy of these agents but also addresses critical challenges associated with traditional wound healing methods, such as prolonged healing times and infection risks. The findings from the literature underscore that ROS play a dual role in wound healing; they are essential for normal physiological processes, including inflammation and angiogenesis, but excessive ROS can lead to tissue damage, too. By utilizing nanomedicine strategies, researchers can fine-tune the levels of ROS at the wound site, promoting a balanced healing environment that accelerates recovery while minimizing oxidative stress. The diverse range of bioactive compounds (e.g., curcumin, resveratrol, and cerium oxide) NPs exhibits significant antioxidant and anti-inflammatory properties. These compounds, when delivered via nanocarriers, enhance cellular responses crucial for tissue regeneration and repair. Therefore, the convergence of bioactive compounds and nanotechnology represents a promising frontier in wound care. Future research should focus on optimizing these formulations for clinical applications, ensuring that they are not only effective but also safe for patients. The potential to revolutionize wound healing protocols through targeted modulation of ROS opens up exciting avenues for therapeutic advancements in regenerative medicine.

## 8. Conclusions

The integration of bioactive compounds into nanomedicine-based carriers offers a promising strategy for modulating ROS and enhancing WH outcomes. Recent advances demonstrate that nanomaterials can be engineered to both scavenge excessive ROS and deliver antioxidants or growth factors, thereby balancing redox states, reducing inflammation, and promoting tissue regeneration. The diverse range of bioactive compounds (e.g., curcumin, resveratrol, etc.) incorporated nanomedicine exhibits significant antioxidant and anti-inflammatory properties. These compounds, when delivered via nanocarriers, enhance cellular responses crucial for tissue regeneration and repair. Innovative approaches such as light-activated nanoclusters and multifunctional nano-scavengers, have shown efficacy in preclinical models by accelerating wound closure, improving collagen deposition, and minimizing scar formation. Despite these encouraging results, further research is required to optimize the safety, efficacy, and clinical translation of these bioactive compound-fortified nanomedicines for routine wound care applications. Therefore, the convergence of nanotechnology and bioactive therapeutics holds significant potential to revolutionize the management of both acute and chronic wounds.

## Data Availability

No new data were created.

## Supplementary Materials

The following supporting information can be downloaded at: https://www.mdpi.com/article/10.3390/pharmaceutics17070855/s1, Table S1: Summary of nanocarriers for wound healing applications. References [115,116,117,126,127,128,129,130,131] are cited in the Supplementary Materials.
