# Correction: Mohite et al. Bioactive Compound-Fortified Nanomedicine in the Modulation of Reactive Oxygen Species and Enhancement of the Wound Healing Process: A Review. *Pharmaceutics* 2025, *17*, 855

**DOI:** 10.3390/pharmaceutics18060648

**Published:** 2026-05-25

**Authors:** Popat Mohite, Abhijeet Puri, Shubham Munde, Nitin Ade, Aarati Budar, Anil Kumar Singh, Deepanjan Datta, Supachoke Mangmool, Sudarshan Singh, Chuda Chittasupho

**Affiliations:** 1AETs St. John Institute of Pharmacy and Research, Palghar 401404, Maharashtra, India; mohitepb@gmail.com (P.M.); abhijeetp@sjipr.edu.in (A.P.); shubhamvmunde@gmail.com (S.M.); nitinade2@gmail.com (N.A.); aaratibudar2000@gmail.com (A.B.); 2Department of Pharmaceutics, United Institute of Pharmacy, Prayagraj 211010, Uttar Pradesh, India; singhanil2682@gmail.com; 3Department of Pharmaceutics, Manipal College of Pharmaceutical Sciences, Manipal Academy of Higher Education, Manipal 576104, Karnataka, India; deepanjandtt@gmail.com; 4Faculty of Pharmacy, Chiang Mai University, Chiang Mai 50200, Thailand; supachoke.man@cmu.ac.th; 5Office of Research Administration, Chiang Mai University, Chiang Mai 50200, Thailand

## Text Correction

There was an error in the original publication [1]. Replacing the content “In a study by Mostafavi et al. on breast cancer cells, quercetin and vitamin C together demonstrated a synergistic effect, reducing Nrf2 expression and inducing oxidative stress within the cancer cells [88]. This study also proposed that these compounds could be used as adjuvants for patients with cancers that overexpress Nrf2. The reduction in fluorescence intensity indicated a significant decrease in ROS, demonstrating quercetin’s effectiveness in combating oxidative stress by scavenging ROS and reducing oxidative damage. These data support quercetin’s antioxidant properties and its potential as a therapeutic agent in reducing oxidative stress in cancer cells [88]. ” to the following: 

“According to L. Zhang et al. [88], non-communicable diseases (NCDs) exhibit a significant correlation with oxidative stress and increased reactive oxygen species (ROS). Quercetin, a flavonoid found in nature, reduces the levels of ROS and has strong antioxidant capabilities. It instigates the Nrf2 signaling pathway, resulting in an enhanced defense against oxidative damage within the body. Quercetin also enhances mitochondrial function and mitigates inflammation through Nrf2 modulation. Quercetin demonstrates promise as a therapeutic agent for treating NCDs due to these characteristics.”

## References

The original reference [88] has been retracted and its contents are no longer of reference value. Replacing original reference [88]:

88.Mostafavi-Pour, Z.; Ramezani, F.; Keshavarzi, F.; Samadi, N. The role of quercetin and vitamin C in Nrf2-dependent oxidative stress production in breast cancer cells. *Oncol. Lett.* **2017**, *13*, 1965–1973.

After corrected, the Reference [88] became as follows:

88.Zhang, L.; Xu, L.-Y.; Tang, F.; Liu, D.; Zhao, X.-L.; Zhang, J.-N.; Xia, J.; Wu, J.-J.; Yang, Y.; Peng, C.; et al. New perspectives on the therapeutic potential of quercetin in non-communicable diseases: Targeting Nrf2 to counteract oxidative stress and inflammation. * J. Pharm. Anal.* **2024**, *14*, 100930. https://doi.org/10.1016/j.jpha.2023.12.020.

The authors state that the scientific conclusions are unaffected. This correction was approved by the Academic Editor. The original publication has also been updated.

## References

[1] Mohite P., Puri A., Munde S., Ade N., Budar A., Singh A.K., Datta D., Mangmool S., Singh S., Chittasupho C. (2025). Bioactive Compound-Fortified Nanomedicine in the Modulation of Reactive Oxygen Species and Enhancement of the Wound Healing Process: A Review. Pharmaceutics.

