# Supplementary material for: Bioactive Compound-Fortified Nanomedicine in the Modulation of Reactive Oxygen Species and Enhancement of the Wound Healing Process: A Review
Source: Pharmaceutics. 2025 Jun 30;17(7):855. doi: 10.3390/pharmaceutics17070855 (PMC12298030; doi:10.3390/pharmaceutics17070855)
Supplement: Supplementary file 1 [file pharmaceutics-17-00855-s001.zip › pharmaceutics-3690753-supplementary.pdf]

Supplementary Table S1. Summary of nanocarriers for wound healing applications.

Type of Nanocarrier	Cytotoxicity threshold (μg/mL)	Remark/Application	Reference
Silver NPs in hydrogel	50–100	The AgNP-loaded hydrogels reduce wound size compared to uncoated injuries.	[126]
Copper NPs in hydrogel	-	Improves antibacterial and antioxidant activity.	[117]
Cerium oxide nanoparticles	50–125	Reversible redox cycling for sustained ROS scavenging. Enhances fibroblast proliferation and tissue regeneration.	[127]
PLGA-Quercetin nanoparticles	150	Excellent antioxidant activity. PLGA ensures deep skin penetration.	[128]
Curcumin-solid lipid NPs hydrogel	-	Enhances solubility and bioavailability of curcumin. Hydrogels improve skin adhesion and moisture retention.	[115]
Zein-Based Nanoparticles in nanofibers	-	Self-enhanced Zn hemostatic bioscaffolds deliver Zn^{2+} and zein proteins to nourish cells.	[129]
Red wine extract gold nanoparticles	20–30	Eco-friendly synthesis using plant polyphenols. Provides antioxidant protection with lower cytotoxicity.	[130]
Ficus trijuga-Loaded Lipid Nanocapsules	40	Delivers flavonoids and triterpenoids. Enhances skin permeation and provides sustained bioactive release.	[116]
Moringa oleifera nanoparticles	50–60	Promotes wound contraction and internal tissue growth.	[131]
